# Results from Türkiye: Which Factors Drive Consumers to Buy Organic Food?

**DOI:** 10.3390/foods13020302

**Published:** 2024-01-18

**Authors:** Murat Baş, Meryem Kahriman, Nihan Çakir Biçer, Selda Seçkiner

**Affiliations:** 1 Department of Nutrition and Dietetics, Faculty of Health Sciences, Acıbadem Mehmet Ali Aydınlar University, 34752 Istanbul, Türkiye; meryem.kahriman@acibadem.edu.tr (M.K.); nihancakir@gmail.com (N.Ç.B.); 2 Department of Nutrition and Dietetics, Faculty of Health Sciences, Istanbul Beykent University, 34398 Istanbul, Türkiye; seldaseckiner@beykent.edu.tr

**Keywords:** organic food, purchasing behaviour, consumer preference, attitude, consumer research

## Abstract

The trend towards organic foods as an alternative has recently increased. Several individual, environmental, and behavioural factors can affect this situation. This study was conducted with 1417 participants to examine the factors affecting attitudes, purchase intention (PI), and actual purchasing behaviour towards organic foods. Consequently, a two-part questionnaire was used to query participants’ sociodemographic information and their attitudes and preferences towards organic foods. Data were analysed using multiple regression analysis, Pearson’s correlation, and structural equation modelling. Our findings confirmed that health consciousness, the knowledge of organic foods, subjective norms, perceived price, values (health and safety), nutritional content, naturalness, availability, monetary barriers, risk barriers, and trust affect attitudes towards organic products. These findings indicate that increasing consumers’ knowledge and awareness about organic foods, encouraging their consumption by society, accessibility them in the food market and making them affordable can affect the attitude towards these products. Furthermore, we determined the direct effect of the attitude on actual buying behaviour with the mediating role of PI. Additionally, we noted that marital status, employment status, disease diagnosis in the last 12 months, and the presence of a baby at home affect actual buying behaviour. In conclusion, they can help food marketers target consumers to their sociodemographic status and develop new sales strategies.

## 1. Introduction

Organic foods include food produced, as much as possible, based on natural substances, physical, mechanical, or biological farming methods, or the cultivation of animals in living conditions suitable for their natural behaviour [1]. Organic foods do not contain genetically modified organisms or chemical additives, and chemical fertilizers or pesticides cannot be used to grow their components [2]. Owing to these features, organic products have a lower impact on the environment than conventional products [3,4].

In recent years, different and novel food technologies have been used for improving food production and designing new food structures [5]. However, these have created environmental and health concerns for consumers [4]. These concerns have directed consumers to organic foods as an alternative, and organic food consumption has increased [6]. Additionally, compared with conventional foods, organic fruits and vegetables have higher phenolic content, whereas organic meat and dairy products have higher omega-3 content. It is believed that this situation may be a driving force for consuming these products [7]. Moreover, reducing the prices of organic foods, facilitating their availability and access, and sociodemographic factors affect attitudes towards these foods [8,9,10,11].

Organic foods are gaining global popularity among consumers. The worldwide food market, which was valued at USD 167.85 billion in 2020, is anticipated to grow at a compound annual growth rate, CAGR) of 14.59%, reaching USD 368.94 billion in 2026 [12]. In Türkiye, organic food production began in the 1980s and was run by international purchasers for export commerce without government assistance, in contrast to the history of most other nations where organic production originated as a farmer–consumer-based movement [13]. Initiatives and funds from public and private contributors eventually hastened the growth of organic agriculture [14]. In 2022, the total area of organic plant product production was 214.101,63 Ha, and the annual organic meat production was 106.53 tons [15]. Further, organic foods appear to be a significant alternative for Turkish consumers [16]. According to studies, age, gender, income status, knowledge level, fear of losing health, and accessibility to organic food are the factors affecting organic product consumption in Türkiye [17,18,19].

Determining these variables is crucial as they have a direct impact on organic farming, livestock husbandry, product marketing, and consumption choices. Therefore, in this study, we aimed to examine the effects of sociodemographic factors such as age, gender, income level, marital status, and household size on actual purchasing behaviour for organic foods. We also aimed to evaluate the effects of knowledge of organic foods, subjective norms, perceived price, values, nutritional content, naturalness, availability, monetary and risk barriers, and trust on attitudes towards organic foods.

### 1.1. Literature Review and Hypotheses

Previous studies have investigated the increasing trend towards organic foods and the reasons for this trend [6,20]. Health perception and environmental concerns are the most frequently reported reasons to be effective in turning to these foods [20,21]. Attitudes and knowledge about these foods, health consciousness, subjective norms, price, and accessibility can also affect the consumption of these foods [4,10,11,22]. Moreover, sociodemographic factors, including age, gender, and income status, may be efficient [4,10].

We aimed to investigate how the following variables, which were also examined by Singh and Verma [10] and Gundala and Singh [4], affect the organic food-purchasing decisions of consumers in Türkiye.

#### 1.1.1. Health Consciousness

Consumers are increasingly concerned about their health; therefore, they frequently attempt to purchase foods that satisfy their minds and nourish their bodies to avoid experiences that can harm their health [23]. Recent studies have demonstrated that consumers are growing more health conscious and usually prefer to purchase natural, organic foods [24,25]. Consequently, it is generally believed that organic foods are healthier than conventional foods [26]. The reason why health-conscious consumers frequently prefer organic foods is that, in addition to being safe and healthy, they do not contain chemicals and additives and are environmentally friendly [27]; further, they contain fewer pesticides and more nutrients. Therefore, organic food consumption is acknowledged as being significantly influenced by health consciousness [28].

Consequently, we hypothesize the following:

**H1.**  *Consumers’ health consciousness (HC) affects their attitude towards organic food purchases.*

#### 1.1.2. Knowledge of Organic Foods

Consumer awareness of and familiarity with organic food has a significant impact on their decision to purchase. Organic food knowledge refers to the ability to understand and judge the unique properties and quality of organic foods. This information can be divided into two main categories, including subjective and objective information. While individuals’ impressions of what they know are referred to as subjective knowledge, their actual comprehension of organic food is referred to as objective knowledge. Studies have reported that attitudes towards organic food consumption are favourably correlated with both objective and subjective knowledge [29,30]. Moreover, knowledge about organic food is expected to be a significant factor in organic food product consumption. Consequently, we hypothesize the following:

**H2.**  *Consumers’ knowledge of organic foods (KOF) affects their attitude towards organic food purchases.*

#### 1.1.3. Subjective Norm

The subjective norm (SN) refers to “the perceived social pressure to perform or not to perform the behaviour”. It presents individuals’ perceptions of what the most significant reference individuals or groups, particularly family and friends, perceive as acceptable or unacceptable behaviour [31]. The literature has explored the efficiency of the SN in defining purchasing intention, and the findings are inconsistent. Scalco et al. [32] stated that the SN significantly affects consumers’ intentions to purchase organic foods, whereas Armitage and Conner [33] pointed to a weaker relationship consistent with the theory of planned behaviour. Consequently, we hypothesize the following:

**H3.**  *Subjective norms (SN) affect consumers’ attitudes towards organic food purchases.*

#### 1.1.4. Perceived Price

Organic foods are priced higher than conventional ones, which can affect consumers’ attitudes [10]. Furthermore, Gan et al. [34] drew attention to a similar situation by reporting that high prices negatively affect the purchasing status of organic foods. In contrast, Radman et al. [35] stated that some consumers are willing to pay high prices, and Smith et al. [36] mentioned that high prices affect attitudes towards these foods only to some extent. Considering the conflicting results, we hypothesize the following:

**H4.**  *Perceived price (PP) affects consumers’ attitudes towards organic food purchases.*

#### 1.1.5. Values (Health and Safety)

Individuals have favourable impressions of organic food as they believe that it is safer, more nourishing, and produced naturally without using dangerous chemicals than conventional foods [37,38]. According to studies, the relationship between the security of value and health may be the main driver of organic food purchases in both developed and developing countries [39]. A study conducted in Türkiye confirmed that individuals’ attitudes about organic foods are influenced by both doctor recommendations and the belief that organic foods reduce the risk of disease and affect attitudes towards these foods [16]. Therefore, the following hypothesis is formulated:

**H5.**  *Values (health and safety) (VAL) affect consumers’ attitudes towards organic food purchases.*

#### 1.1.6. Nutritional Content

Organic foods are believed to be more nutritious than conventional foods and are grown naturally without using hazardous chemical fertilizers [38,40,41]. Previous studies have demonstrated that consumers’ growing preference for organic food is largely because of its high nutritious content [42]. Consequently, we hypothesize the following:

**H6.**  *Nutrient content (NC) affects consumers’ attitudes towards organic food purchases.*

#### 1.1.7. Naturalness

Consumers perceive food naturalness as a “decisive purchase incentive”, and most individuals observe a close relationship between “healthy” and “natural” [43]. Studies on organic foods have shown that attitudes towards organic food are related to the perceived significance of naturalness in food [44,45]. Consequently, the following hypothesis is conceptualized:

**H7.**  *Naturalness (NA) affects consumers’ attitudes towards organic food purchases.*

#### 1.1.8. Availability

The lack of easy access to organic foods is an essential factor that drives consumers to purchase traditional foods [46]. Limited availability can negatively affect consumer attitudes and purchasing behaviour towards organic food products. In contrast, purchasing behaviour can be positively affected by easy access to organic food products [47]. Consequently, we hypothesize the following:

**H8.**  *Availability (AVA) affects consumers’ attitudes towards organic food purchases.*

#### 1.1.9. Monetary Barrier

The traditional economic theory perceives price as a monetary value required to ensure the purchase of products. A high price can make consumers realize the economic cost more strongly and have a negative impact on their purchasing intentions [48]. Furthermore, almost half of the consumers stated that they would purchase organic foods if they were more affordable [27]. Therefore, it is believed that price may pose an obstacle not only to purchasing but also to repeating organic food purchases. Consequently, we hypothesize the following:

**H9.**  *The monetary barrier (MB) affects consumers’ attitudes towards organic food purchases.*

#### 1.1.10. Risk Barrier

Several individuals are unaware of what the phrases organic, certification system, labelling on products, and how they define an organic product actually imply. The low rate of organic purchasing could be partly because of this lack of understanding [49]. Therefore, evaluating the effects of consumers’ awareness of the organic food term, labelling, and certification on their purchasing behaviour is necessary. Consequently, we hypothesize the following:

**H10.**  *The risk barrier (RB) affects consumers’ attitudes towards organic food purchases.*

#### 1.1.11. Trust (Overall)

As organicity cannot be detected by consumers who purchase products, organic fraud can be difficult to notice. Moreover, it requires consumers to trust the food chain members responsible for certifying products. Despite greater control and certification, organic products remain considered more vulnerable to authenticity and counterfeiting concerns than traditional products [50]. Consumers’ confidence in the social performance of manufacturers and retailers also has critical effects on their purchasing behaviour [51,52]. In conclusion, owing to the difficulty and ambiguity of certification processes, consumers’ perceptions of and trust in organic food are difficult to evaluate. Consequently, we hypothesize the following:

**H11.**  *Trust (TR) affects consumers’ attitudes towards organic food purchases.*

#### 1.1.12. Purchase Intention

Companies utilize the marketing literature’s dimension of intention to forecast the sales of new items or the repurchase of old products [53]. Several variables, including perceptions of health, environmental awareness, accessibility, quality, nutritional content, and product distribution, can influence purchase intention (PI) for organic foods [2]. Similarly, in Türkiye, it has been reported that different reasons, including the significance of environmental pollution, respect for ecological selectivity, and providing significance to the origin of food, affect attitudes towards these foods [16]. Actual purchasing behaviour emerges as a result of the interaction of intention and willingness, as reported by Ajzen [54]. Consequently, we hypothesize the following:

**H12.**  *Consumers’ attitudes towards organic foods positively affect their PI.*

#### 1.1.13. Actual Buying Behaviour

Increasing organic food-purchasing behaviour is closely related to a positive attitude and PI. Ajzen [54] stated that “behaviour is a function of adaptive intentions and behavioural control”. Comprehending how consumers perceive organic foods is significant as this affects whether or not they intend to purchase the products. Additionally, this will influence how individuals actually purchase food [55]. This discrepancy between the actual purchasing behaviour of organic products and the favourable attitude of consumers is called the “attitude–behaviour difference”. Singh and Verma [10] mentioned that consumers’ favourable attitudes towards organic foods do not necessarily translate into action. Determining why favourable attitudes have a weaker effect on the actual purchase of organic food products and PI is significant. Consequently, we hypothesize the following:

**H13.**  *PI positively affects actual buying behaviour towards organic foods.*

**H14.**  *Attitudes towards organic foods directly impact actual buying behaviour through PI mediation.*

#### 1.1.14. Sociodemographic Factors

##### Age

The relationship between age and organic food consumption has different results. Recent studies have reported that organic food consumers are usually middle-aged or older adults [56,57]. However, Magnusson et al. [58] and Dettmann and Dimitri [59] stated that younger customers are more interested in organic foods, whereas older adult consumers do not consistently purchase organic items.

##### Gender

Most of the studies have reported that women tend to be more worried about the environment and ecological problems; therefore, they are more inclined to consume organic foods. Conversely, the tendency of women to consume more organic food than men is associated with higher awareness of food safety and environmental problems [39,60,61].

##### Education

Several studies have shown that education level is positively associated with organic food consumption [57,62,63]. However, some studies have emphasized the negative effect of education level on organic food consumption [64,65].

##### Marital Status

Married women can purchase more organic foods owing to their food shopping role [60]. In contrast, other studies have indicated that consumers without partners are more likely to purchase organic food products [66,67].

##### Having Children and Household Size

Several studies have shown that houses with children under the age of 18 consume more organic food, families with children are more inclined to purchase organic food, and there is a significant demand for organic baby food [27,39,65]. In addition to having children, household size is also a controversial issue for organic food demand. In contrast to other studies, Tsakiridou et al. [68] stated that household size is not a significant determinant of demand for organic foods. Harris et al. [66] reported that the demand for organic foods is negatively correlated with household size.

##### Employment Status

There is limited research in the literature investigating how occupation affects the demand for organic foods. Chen et al. [69] and Xie et al. [57] mentioned that office employees constitute most of Chinese organic food consumers. Studies conducted in Türkiye reported that unemployment has a significant role in the low demand for organic foods, and public officials and housewives comprise most organic product consumers [70,71].

##### Income

As organic foods cost more than conventional foods, purchasing decisions are influenced by income. In addition to studies reporting the relationship between income and organic food demand [61,72], there are also those claiming that there is no specific determinant for organic demand [66]. Conversely, in Türkiye, Oraman [73] noted that organic product consumers have above-average incomes.

Consequently, we hypothesize about sociodemographic factors as follows:

**H15a.**  *A significant difference exists according to the age variable for the actual purchasing behaviour of organic food products.*

**H15b.**  *A significant difference exists according to the gender variable for the actual purchasing behaviour of organic food products.*

**H15c.**  *A significant difference exists according to the education variable for the actual purchasing behaviour of organic food products.*

**H15d.**  *A significant difference exists according to the marital status for the actual purchasing behaviour of organic food products.*

**H15e.**  *A significant difference exists in the actual purchasing behaviour of organic food products according to the status of having children.*

**H15f.**  *A significant difference exists according to the household size variable for the actual purchasing behaviour of organic food products.*

**H15g.**  *A significant difference exists according to the occupation variable for the actual purchasing behaviour of organic food products.*

**H15h.**  *A significant difference exists according to the income variable for the actual purchasing behaviour of organic food products.*

**H15i.**  *A significant difference exists in the actual purchasing behaviour of organic food products according to the status of having been diagnosed with a disease in the last 12 months.*

## 2. Materials and Methods

### 2.1. Sample and Data Collection Procedure

This study was conducted online through a survey prepared using Google Forms and included participants over the age of 18 who volunteered to participate in this study. The sample size was calculated as 342 using the G*Power program (version 3.1), considering the studies in the literature, with α = 0.05 margin of error and 95% test power [4,10]. Participants were reached through social media platforms. They were asked to read and approve an informed consent form before study initiation. Furthermore, before study initiation, we received ethical approval from the Acıbadem Mehmet Ali Aydınlar University Medical Research Ethics Committee (ATADEK-2023/13, 17 August 2023). This study was conducted in accordance with the Declaration of Helsinki, and all participants were asked to approve the informed consent form.

Using a two-part questionnaire (Supplementary File S1), the proposed hypotheses were tested. In the first section, participants’ sociodemographic information was gathered, including their age, gender, occupation, education level, household size, income level, marital status, having children, and factors affecting the decision to purchase organic foods. In the second part, questions were asked to evaluate the attitudes and preferences of consumers towards organic foods. The items in the questionnaire were adopted from previous studies [10,22]. The 38 items were scored on a 5-point Likert scale ranging from “Strongly Disagree” to “Strongly Agree”.

### 2.2. Statistical Analysis

Descriptive statistics for categorical variables are stated as percentages and frequencies. To determine if the numerical variables adhered to the normal distribution, the Shapiro–Wilk test was used. For data with a normal distribution, the descriptive statistics for numerical variables were presented as means and standard deviations (SDs), and for data without a normal distribution, as median (minimum–maximum) values. Convergent validity was considered for the construct validity of the scale [74]. All composite reliability values for the scale are expected to be higher than average variance extracted (AVE) values; to ensure convergent validity, the AVE value is expected to be more than 0.5. Additionally, the standardized factor loads of the items should be above 0.5, and the combined reliability value should be higher than 0.7 [74]. The AVE value is obtained by dividing the sum of the squares of the item loads of the AVE factor by the number of expressions [75]. To determine the reliability level of the scale, Cronbach’s alpha coefficient was calculated. For discriminant validity, two new values must be calculated. To provide discriminant validity, meeting the conditions, including maximum shared variance (MSV) < AVE, average shared variance (ASV) < MSV, and the square root of the AVE being greater than the correlation between the factors, is necessary [76]. Structural equation modelling was used for mediation analysis. Structural equation modelling analyses were performed using the Lavaan and Sem packages of the R Project v3.6.1 (R Core Team, Vienna, Austria) software [77]. The relationships between the scales were investigated using Pearson’s correlation coefficient for data showing normal distribution. To test the effect between variables, multiple regression analysis was used. In all calculations and interpretations, the statistical significance level was considered as “*p* < 0.05”, and the hypotheses were established as bidirectional. All statistical analyses were performed using the Statistical Package for the Social Sciences (version 26, IBM Inc., Chicago, IL, USA) and R Project v3.6.1 (R Core Team, Vienna, Austria) package program.

## 3. Results and Discussion

### 3.1. Description of the Sample

The demographic and organic food consumption characteristics of participants are presented in Table 1. Of the participants, 72.2% were women (*n* = 1023), and their mean age was 34.14 ± 11.58 years. The results showed that 51.1% of the participants (*n* = 724) had a bachelor’s degree, and 34.4% of the participants (n = 487) had an income between 20,001 and 40,000 Turkish Liras (TRY. Furthermore, 55.1% of the participants (n = 781) lived in families of 3–4 individuals. Moreover, 9.7% of the participants (n = 137) had a baby in the last 12 months.

### 3.2. Reasons for Purchasing Organic Foods

**Figure 1:**
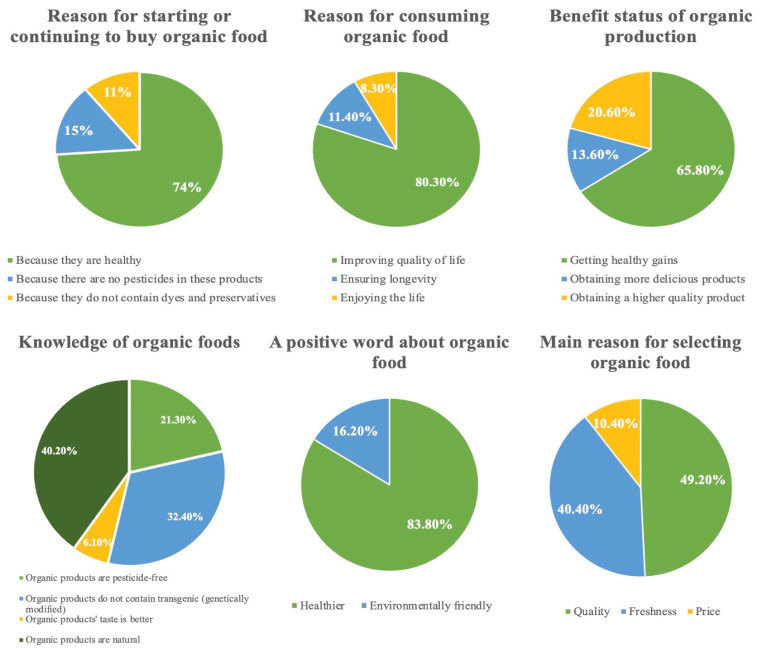
Factors affecting consumers’ organic food consumption preferences.

Of the participants, 24.4% (*n* = 346) reported purchasing organic fruits and vegetables regularly. Additionally, 74% of the participants (*n* = 1048) preferred organic food as it is healthy, 80.4% (*n* = 1139) consumed organic food as it improves their quality of life, 65.8% (*n* = 933) believed that organic production is beneficial as it provides healthy gains, and 49.3% (*n* = 698) expressed that their main reason for selecting organic food was quality (Table 1) (Figure 1). When organic food information was asked, 40.2% of the participants (*n* = 570) stated that organic products are natural. Our findings, particularly emphasizing that the participants consume organic food for health, supported those of previous studies [4,10,22].

It was observed that the standardized factor loads of the items were between 0.626 and 0.982, the CR and Cronbach’s alpha values were higher than 0.7, the AVE values were >0.5, and all of the 16 constructs had convergent validity. Cronbach’s alpha values ranging from 0.70 to 0.79 indicated acceptable reliability [78]. Moreover, the minimum acceptable factor load was 0.40 [79]. Therefore, these results indicated that the scale used in the research had reliability and convergent validity (Table 2).

When the discriminant validity of the study variables was examined, AVE values were lower than the corresponding CR values, and all ASV values were lower than the MSV values. Based on these findings, it was determined that discriminant validity was provided. Additionally, significant correlations were determined between all study variables (*p* < 0.001) (Supplementary File S2).

**Figure 2:**
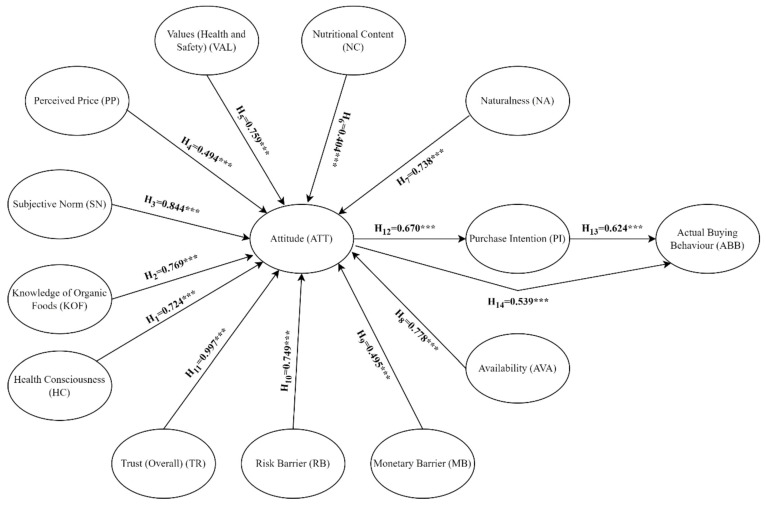
Tested model results. (*** *p* < 0.001).

All study variables affected the attitude variable (*p* < 0.001), and the effect was direct. Health awareness, KOF, SN, PP, values (health and safety), nutritional content, naturalness, availability, MB, RB, and trust (general) scores had an effect of 72.4%, 76.9%, 84.4%, 49.4%, 75.9%, 40.4%, 73.8%, 77.8%, 49.5%, 74.9%, and 99.7%, respectively, on the attitude scores (Table 3, Figure 2). Similarly, in the studies conducted by Gundala and Singh [4] in the United States and by Singh and Verma [10] in India, it has been reported that KOF, health consciousness, PP, and subjective norms affect consumers’ attitudes towards organic foods. Tandon et al. [11], on the other hand, reported that risk-related barriers can foster a positive attitude towards organic foods. In our study, it has been confirmed that the risk barriers faced by consumers can positively affect their attitude towards these foods. Similarly, our findings showed that the monetary barrier positively affects attitude. Another finding that supported this was that perceived price had a positive effect on attitude. Radman et al. [35] pointed out that some consumers are willing to purchase high-priced products. These findings suggest that the risk-related barriers and monetary barriers faced by consumers may be the driving forces that may direct consumers to organic foods. In another study conducted in Vietnam, it was emphasized that values (health and safety consciousness) and trust are associated with favourable attitudes [22]. These findings in the literature indicate that although there are geographical differences, these variables can affect consumers’ attitudes. Our findings highlighted that health consciousness, KOF, subjective norms, values (health and safety), PP, nutritional content, naturalness, MB, RB, availability, and trust affect attitude towards organic food, and H1–H11 were confirmed.

**Figure 3:**
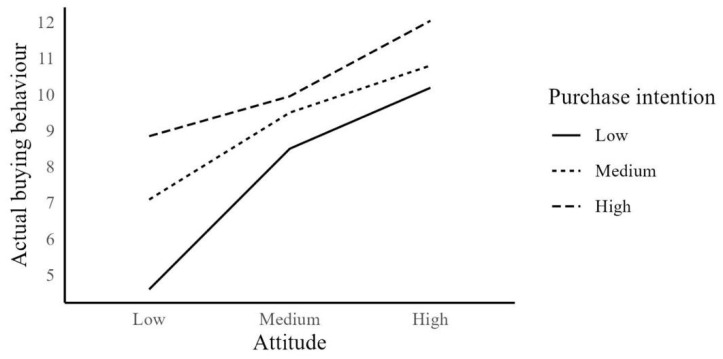
Moderator effect of purchase intention on attitude and purchasing behaviour.

It was believed that besides the effect of environmental and personal factors on attitude towards organic foods, attitude also increases PI [80]. Therefore, we evaluated the effect of attitude on PI and actual buying behaviour (ABB). The attitude scores had an effect of 67% on PI scores (*p* < 0.001) and a 62.4% effect on PI scores on ABB scores (*p* < 0.001). Additionally, the attitude variable, wherein the PI variable played a mediating role, had a direct effect on the ABB variable (*p* < 0.001) (Table 4, Figure 3). Moreover, participants with high PI had higher ABB for their changing attitudes, whereas those with low PI had lower ABB (Figure 3). Furthermore, Gundala and Singh [4] stated that attitude and PI affect ABB with their mediation effect. Similarly, another study by Singh and Verma [10] supported similar findings. Conversely, Parashar et al. [80] highlighted a positive relationship between PI and actual purchasing and confirmed that attitude played a moderator role in this relationship. The literature and our findings confirm the relationship between attitude and PI and their positive effects on ABB (H12–H14).

**Figure 4:**
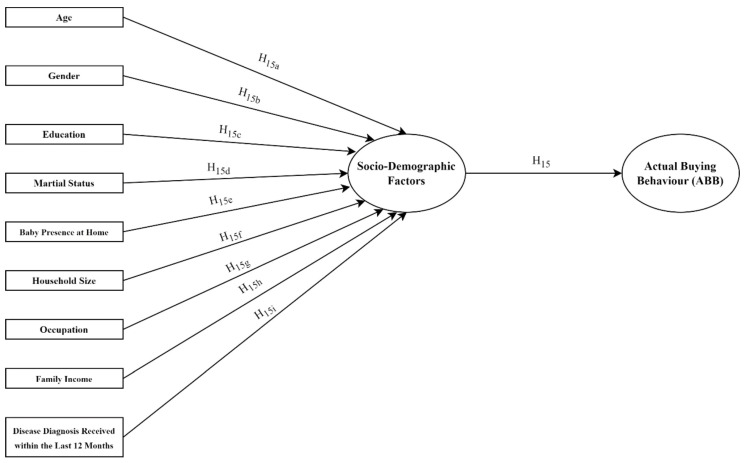
Multiple regression analysis with sociodemographic variables.

The variables of gender, age, education level, family income level, and family size did not have a significant effect on ABB scores (*p* > 0.05). Previous studies reported that gender, age, education, income status, and household size affect organic purchasing [4,10,57,66], which were not consistent with our findings. However, marital status, employment status, disease diagnosis in the last 12 months, and presence of a baby at home had a statistically significant effect (*p* < 0.05 and *p* < 0.01) on ABB scores. The ABB of single individuals compared with married individuals and of non-working individuals compared with working individuals was higher at a rate of 50.4% and 42.7%, respectively. Although there are studies in the literature stating that single individuals purchase more organic products [66,67,81], there are also studies that refer to the positive role of marriage in this regard [60,82,83]. Furthermore, previous studies in Türkiye reported that unemployment is a significant limiter for the demand for organic products [70,71]. Therefore, our findings are surprising. Additionally, the ABB of individuals who were not diagnosed with the disease in the last 12 months was 63.5% less than that of individuals who were diagnosed. Furthermore, the ABB of individuals who did not have a baby at home were 78.2% less than those who had a baby at home (Table 5) (Figure 4). Families with children, particularly those under the age of 18, purchase more organic foods [27,39]. Our findings demonstrated that some but not all demographic factors influence organic product purchasing behaviour and confirmed H15d, H15e, and H15g.

## 4. Conclusions

Here, we aimed to examine consumers’ reasons for purchasing organic foods, the effects of different variables on attitudes towards these foods, and the effects of sociodemographic factors on ABB. We determined that efforts to improve the quality of life and health awareness are the most significant reasons for turning to organic foods. Previous studies reported that attitude and PI towards these foods do not frequently result in ABB. Therefore, we evaluated the effects of different variables on attitude and ABB. We noted that health consciousness, KOF, values, PP, subjective norms, nutritional content, naturalness, availability, MB, RB, and trust have a direct effect on attitude. These findings show that accessibility in the food market, increasing consumers’ knowledge and awareness about these products, encouraging the consumption of these foods by society and marketing them at affordable prices can affect attitudes. Further, we confirmed that attitude affects ABB, with the mediating role of PI. Moreover, marital status, employment status, disease diagnosis in the last 12 months, and presence of a baby at home affected ABB. These findings may guide organic food marketers. It can enable them to distinguish consumers according to sociodemographic factors and understand the factors affecting organic food consumption, thereby helping them develop their marketing strategies. 

### Limitations of This Study

This study had some limitations. This study was conducted online, and the findings represented only a particular group. Therefore, the findings should be evaluated with this situation in mind. Additionally, the effects of only some factors were examined. Studies with larger samples that also evaluate other factors should be conducted.

## Figures and Tables

**Table 1:** Demographic and organic food consumption characteristics of participants.

	*n*	%
Gender		
Men	394	27.8
Women	1.023	72.2
Age (year) (mean ± SD)	34.14 ± 11.58	
Education level		
Primary school graduate	28	2.0
High school graduate	300	21.2
Associate degree	138	9.7
Bachelor’s degree	724	51.1
Postgraduate	227	16.0
Marital status		
Married	644	45.4
Single	773	54.6
Employment status		
Student	394	27.8
Private sector employee	395	27.9
Self-employment	200	14.1
Housewife	107	7.6
Public servant	183	12.9
Unemployed	79	5.6
Retired	59	4.2
Family income level		
TRY 20,000 and below	477	33.6
Between TRY 20,001 and 40,000	487	34.4
TRY 400,001 and above	453	32.0
Disease diagnosis in the last 12 months		
Yes	227	16.0
No	1.190	84.0
Household size		
Between 1 and 2 individuals	470	33.2
Between 3 and 4 individuals	781	55.1
5 individuals and above	166	11.7
Baby presence at home in the last 12 months		
Yes	137	9.7
No	1.280	90.3
Purchase status of organic fruit and vegetables in the last month		
Yes, I regularly buy organic vegetables and fruits	346	24.4
Yes, I bought it once last month	119	8.4
Yes, I bought it twice last month	128	9.0
Yes, I bought it three times last month	91	6.4
No	733	51.7
Frequency of purchase of organic food		
I have never bought	733	51.7
I bought one organic product	124	8.8
I bought two organic products	165	11.6
I bought three organic products	106	7.5
I bought four or more organic products	289	20.4
Changes in organic food consumption in the last 12 months		
My organic food consumption has not changed	610	43.0
I made no effort to buy organic food	334	23.6
My organic food consumption has decreased	206	14.5
My organic food consumption has increased	267	18.8

TRY, Turkish Liras.

**Table 2:** Reliability and convergent validity.

Constructs	Items	Standardized Factor Loadings	CR	AVE	Cronbach’s Alpha
Health consciousness (HC)	HC1	0.887	0.896	0.743	0.826
	HC2	0.884			
	HC3	0.812			
Knowledge of organic foods (KOF)	KOF1	0.823	0.886	0.723	0.805
	KOF2	0.812			
	KOF3	0.897			
Subjective norm (SN)	SN1	0.893	0.888	0.727	0.812
	SN2	0.897			
	SN3	0.761			
Perceived price (PP)	PP1	0.935	0.933	0.874	0.855
	PP2	0.935			
Purchase intention (PI)	PI1	0.885	0.913	0.777	0.855
	PI2	0.844			
	PI3	0.914			
Actual buying behaviour (ABB)	ABB1	0.856	0.898	0.745	0.829
	ABB2	0.886			
	ABB3	0.847			
Attitude (ATT)	ATT1	0.656	0.906	0.767	0.828
	ATT2	0.967			
	ATT3	0.967			
Values (health and safety) (VAL)	VAL1	0.982	0.985	0.957	0.978
	VAL2	0.974			
	VAL3	0.979			
Nutritional content (NC)	NC1	0.914	0.910	0.835	0.803
	NC2	0.914			
Naturalness (NA)	NA1	0.626	0.899	0.754	0.822
	NA2	0.967			
	NA3	0.967			
Availability (AVA)	AVA1	0.879	0.962	0.893	0.939
	AVA2	0.977			
	AVA3	0.976			
Monetary barrier (MB)	MB1	0.879	0.872	0.773	0.707
	MB2	0.879			
Risk barrier (RB)	RB1	0.875	0.858	0.669	0.748
	RB2	0.776			
	RB3	0.799			
Trust (overall) (TR)	TR1	0.845	0.887	0.664	0.830
	TR2	0.861			
	TR3	0.713			
	TR4	0.833			

AVA, availability; ABB, actual buying behaviour; ATT, attitude; AVE, average variance extracted; CR, composite reliability; HC, health consciousness; KOF, knowledge of organic foods; MB, monetary barrier; NA, naturalness; NC, nutritional content; RB, risk barrier; SN, subjective norm; PI, purchase intention; PP, perceived price; VAL, values (health and safety); TR, trust (overall).

**Table 3:** Effects of study variables on the attitude variable.

	*β*	t-Value	*p*-Value
HC→ATT	0.724	15.801	<0.001 ***
KOF→ATT	0.769	16.675	<0.001 ***
SN→ATT	0.844	17.931	<0.001 ***
PP→ATT	0.494	15.609	<0.001 ***
VAL→ATT	0.759	16.552	<0.001 ***
NC→ATT	0.404	13.868	<0.001 ***
NA→ATT	0.738	16.862	<0.001 ***
AVA→ATT	0.778	15.992	<0.001 ***
MB→ATT	0.495	16.019	<0.001 ***
RB→ATT	0.749	16.367	<0.001 ***
TR→ATT	0.997	16.051	<0.001 ***

AVA, availability; ATT, attitude; HC, health consciousness; KOF, knowledge of organic foods; MB, monetary barrier; NA, naturalness; NC, nutritional content; RB, risk barrier; SN, subjective norm; PP, perceived price; VAL, values (health and safety); TR, trust (overall). β, beta coefficient; *** *p* < 0.001.

**Table 4:** Effect of the attitude variable on the purchase intention variable, effect of the purchase intention variable on the actual buying behaviour variable, and effect of the attitude variable on the actual buying behaviour variable, with the purchase intention variable in the mediating role.

	β	t-Value	*p*-Value
ATT→PI	0.670	14.986	<0.001 ***
PI→ABB	0.624	14.723	<0.001 ***
ATT→PI→ABB	0.539	22.744	<0.001 ***

ABB, actual buying behaviour; ATT, attitude; PI, purchase intention. β, beta coefficient; *** *p* < 0.001.

**Table 5:** Effect of demographic variables on the actual buying behaviour variable.

	Unstandardized Coefficients				95.0% Confidence Interval for B	
	β	SE	t	*p*	Lower Bound	Upper Bound
(Constant)	10.680	0.607	17.583	<0.001 ***	9.489	11.872
Gender (ref: women)						
Men	−0.014	0.193	−0.071	0.943	−0.392	0.365
Age (year)	0.014	0.010	1.503	0.133	−0.004	0.033
Marital status (ref: married)						
Single	0.504	0.228	2.216	0.027 *	0.058	0.951
Education level (ref: bachelor and above)						
High school and below	−0.378	0.219	−1.720	0.086	−0.808	0.053
Associate degree	−0.122	0.294	−0.415	0.678	−0.700	0.455
Family income (Ref: TRY 40,001 and above)						
TRY 20,000 and below	0.024	0.218	0.109	0.913	−0.404	0.451
Between TRY 20,001 and 40,000	−0.171	0.210	−0.815	0.415	−0.583	0.241
Household size	−0.009	0.068	−0.127	0.899	−0.142	0.125
Occupation (ref: worker)						
Nonworker	0.423	0.192	2.207	0.027 *	0.047	0.799
Disease diagnosis received within the last 12 months (ref: yes)						
No	−0.635	0.238	−2.664	0.008 **	−1.102	−0.167
Baby presence at home (ref: yes)						
No	−0.782	0.298	−2.628	0.009 **	−1.366	−0.198

TRY, Turkish Liras; β, beta coefficient; SE, standard error; t, independent samples *T*-test; * *p* < 0.05; ** *p* < 0.01; *** *p* < 0.001.

## Data Availability

Data is contained within the article or supplementary material.

## Supplementary Materials

The following supporting information can be downloaded at: https://www.mdpi.com/article/10.3390/foods13020302/s1, Supplementary File S1: Questionnaire Used In The Study; Supplementary File S2: Correlation Coefficients Between Variables.
