# Supplementary material for: Results from Türkiye: Which Factors Drive Consumers to Buy Organic Food?
Source: Foods. 2024 Jan 18;13(2):302. doi: 10.3390/foods13020302 (PMC10815511; doi:10.3390/foods13020302)
Supplement: Supplementary file 1 [file foods-13-00302-s001.zip › foods-2775693-supplementary.pdf]

Supplementary File 1

QUESTIONNAIRE USED IN THE STUDY

1. Sociodemographic Information

1. Gender
- o Female
- o Male

2. Age (Please write your year of birth. For example: 1970)
…………..
3. Weight (kg) (Please write as a whole number. For example: 85)
…………..
4. Height (cm) (Please write in cm, such as 182)
…………..
5. Your Education Level:
o Primary school-secondary school graduate
o High school graduate
o Associate's degree graduate
o Bachelor's degree
o Postgraduate (master's / doctorate / specialization)
6. Your marrital status:
o Single
o Married
7. Your employment status:
   - o Housewife
   - o Private sector employee
   - o Civil servant
   - o Student
   - o Retired
   - o Self-Employment
   - o Unemployed/Not working
   - o Employer
8. Your Individual Income (Monthly):
o Below minimum wage
o Around minimum wage
o 5,500 - 10,000 TL

o 10,000 - 15,000 TL

o Over 15,000 TL

o I am a student and I receive pocket money from my family.

9. Your Family Income (Monthly):

o Below minimum wage

o Around minimum wage

o 5,500 - 10,000 TL

o 10,000 - 15,000 TL

o 15,000-20,000 TL

o 20,000-25,000 TL

o 25,000-30,000 TL

o 30,000-35,000 TL

o 35,000-40,000 TL

o 40,000 TL and above

10. Have you had a new disease diagnosed by a doctor in the last 12 months?
    - o Yes
    - o No
11. Please mark any new disease(s) diagnosed by a doctor in the last 12 months.
    - o Obesity
    - o High Blood Pressure
    - o Musculoskeletal diseases
    - o Hormonal diseases
    - o Cancer
    - o Cardiovascular diseases
    - o Diabetes
    - o Respiratory system diseases
    - o Digestive system diseases (liver, gallbladder, stomach, etc.)
    - o Vitamin and mineral deficiencies (iron, vitamin B12 deficiency, etc.)
    - o Mental problems (depression, overeating, vomiting, night eating, etc.)
    - o High blood lipids (such as LDL cholesterol, total cholesterol, triglycerides)
    - o Other
12. Do you smoke?
    - o Yes
    - o No

13. Do you consume alcohol regularly?
    - Yes
    - No
14. Do you exercise at least 150 minutes a week (such as brisk walking, running)?
    - Yes
    - No
15. How many people live in the house, including you?

- 1
- 2
- 3
- 4
- 5
- 6
- 7 and above

16. Which of the following describes you?

- I live alone
- I live with a husband/wife/partner and we have no children
- I live with my husband/wife/partner and we have a child
- I am not married and I live with family members
- I live with my housemates
- I am single and I live with my child/children

17. Has anyone had a baby in your household in the last 12 months (you or a family member you live with may have a baby)
    - Yes
    - No
18. Have you personally bought organic fruits and vegetables in the last month?
    - No
    - Yes, I bought it once last month
    - Yes, I bought it twice last month
    - Yes I bought it 3 times last month
    - Yes, I regularly buy organic vegetables and fruits
19. How many organic food products did you buy last month (for example, packaged products other than vegetables and fruits such as organic tomato paste, jam, honey)?
    - I never received

- I bought that organic product
- I bought 2 organic products
- I bought 3 organic products
- I bought 4 or more organic products

20. Has there been a change in your organic food consumption in the last 12 months?
    - My consumption of organic food has increased
    - My consumption of organic food has decreased
    - My organic food consumption has not changed
    - I did not try to buy organic food
21. What is the main reason to start or continue BUYING ORGANIC FOOD?

    Because there are no agricultural pesticides in these products

    Because it does not contain dyes and preservatives

    Because they are healthy
22. Which of these values can be associated with ORGANIC FOODS?
    - Longevity
    - Enjoying life
    - Quality of life
23. What are the consequences of ORGANIC FOOD consumptıon?
    - Getting healthy gains
    - Obtaining a higher quality product
    - Obtaining more delicious products
24. What are the characteristics associated with ORGANIC FOODS?
    - Organic products are more natural
    - Organic products do not contain transgenic (genetically modified)
    - Organic products do not contain pesticides
    - Organic products' tastes better
25. In your opinion, what words create a positive image for ORGANIC FOOD?
    - More healthy
    - Eco-friendly
26. What is your main selectıon criteria when buying organic foods?
    - Quality
    - Price
    - Freshness
27. Which ORGANIC PRODUCTS do you usually consume?

- Red meat
- Fruits
- Vegetables
- Egg
- Cereals (such as quinoa, buckwheat, bulgur)
- Legumes (such as chickpeas, beans)
- Milk
- Chicken

28. Which one describes your eating habits in the last 12 months?
    - My eating habits have changed positively
    - My eating habits have changed negatively
    - There was no change in my eating habits

2.Questionnaire for Evaluation of Consumers' Attitudes and Choices Towards Organic Food

I'm thinking of buying organic food.	The items in the questionnaire were scored on a 5-point Likert-type scale ranging from 'Strongly Agree' to 'Strongly Disagree'.
I am very likely to buy organically processed food.	
I'm very likely to buy organic food.	
I try to buy organic food as it is the best choice for me.	
Despite the high prices, I am willing to buy organic food.	
Health threats can be prevented by consuming organic food.	
Consuming organic food is effective in preventing health problems.	
If I consume organic food, I am less likely to take health risks.	
Consuming organic food can enrich my nutrition level.	
Consuming organic food keeps me healthy for a long time.	
I can express my concern for the environment by buying organic food.	
By buying organic food, I can show myself and my friends th at I care about protecting the environment.	
My friends can perceive my interest in environmental issues when buying organic food.	
People around me observe that I am aware of ecological deve lopment when buying organic food.	
My concern about chemical hazards when buying organic fo od is noticed by people.	
I prefer organic foods because they are processed without the use of any chemicals.	
I prefer organic foods because they are more nutritious than non-organic foods.	
I prefer organic foods because they cause less disease than co nventional foods.	
I prefer organic food because it is environmentally friendly.	
I prefer organic foods as no preservatives are used to extend t he shelf life.	
People whose opinions I value prefer me to buy organic food.	
My interaction with people regarding organic consumables influences me to buy organic food.	
My friends approve of my decision to buy organic food.	
My close friends and family members will be happy if I buy organic food.	
People around me generally believe that using organic food is better for our health.	
I'm sure I could buy organic food if I wanted.	
I have enough resources and time to buy organic food.	
Whether or not I buy organic food is entirely up to me.	
I pay a high price for organic food because my health will be protected.	
I am someone who knows organic products well.	
There are organic foods in the places where I shop.	
Buying organic food instead of conventional food makes me feel better.	
I'm willing to pay more for food that doesn't harm the enviro nment.	
I am a person who thinks about my health a lot.	
Individuals should pursue their goals only after considering society.	

My consumption decisions are influenced by external factors (family, friends, etc.).	
I have full confidence in the certification/origin of organic foods.	
I am a person aware of my environmental responsibilities.	

Supplementary File 2: Correlation Coefficients Between Variables

		HC	KOF	SN	PP	VAL	NC	NA	AVA	MB	RB	TR	ATT	PI
**KOF**	**r**	0.636***												
**SN**	**r**	0.566***	0.676***											
**PP**	**r**	0.608***	0.777***	0.578***										
**VAL**	**r**	0.446***	0.552***	0.691***	0.455***									
**NC**	**r**	0.498***	0.399***	0.481***	0.342***	0.445***								
**NA**	**r**	0.548***	0.617***	0.722***	0.519***	0.670***	0.424***							
**AVA**	**r**	0.565***	0.624***	0.557***	0.561***	0.468***	0.408***	0.488***						
**MB**	**r**	0.617***	0.600***	0.681***	0.524***	0.653***	0.582***	0.634***	0.512***					
**RB**	**r**	0.584***	0.605***	0.725***	0.530***	0.674***	0.503***	0.680***	0.578***	0.768***				
**TR**	**r**	0.650***	0.624***	0.621***	0.582***	0.490***	0.407***	0.569***	0.672***	0.534***	0.710***			
**ATT**	**r**	0.637***	0.720***	0.792***	0.615***	0.626***	0.496***	0.663***	0.617***	0.679***	0.718***	0.677***		
**PI**	**r**	0.614***	0.576***	0.511***	0.511***	0.421***	0.465***	0.465***	0.544***	0.584***	0.498***	0.541***	0.569***	
**ABB**	**r**	0.585***	0.605***	0.685***	0.547***	0.619***	0.460***	0.607***	0.537***	0.632***	0.659***	0.759***	0.659***	0.555***

HC: Health Consciousness; KOF: Knowledge of Organic Food; SN: Subjective Norm; PP: Perceived Price; PI: Purchase Intention; ABB: Actual Buying Behaviour; ATT: Attitude; VAL: Values (Health and Safety); NC: Nutritional Content; NA: Naturalness; AVA: Availability; MB: Monetary Barrier; RB: Risk Barrier; TR: Trust (Overall)

r: Spearman Correlation Coefficient

***$p<0.001$
